# Fiber-Embedded Metallic Materials: From Sensing towards Nervous Behavior

**DOI:** 10.3390/ma8115435

**Published:** 2015-11-24

**Authors:** Nouari Saheb, Samir Mekid

**Affiliations:** 1 Mechanical Engineering Department, King Fahd University of Petroleum and Minerals, Dhahran 31261, Saudi Arabia; smekid@kfupm.edu.sa; 2 Center of Research Excellence in Nanotechnology, King Fahd University of Petroleum and Minerals, Dhahran 31261, Saudi Arabia

**Keywords:** metals, embeddable fibers, embedding processes, smart materials, nervous materials, structural health monitoring

## Abstract

Embedding of fibers in materials has attracted serious attention from researchers and has become a new research trend. Such material structures are usually termed “smart” or more recently “nervous”. Materials can have the capability of sensing and responding to the surrounding environmental stimulus, in the former, and the capability of feeling multiple structural and external stimuli, while feeding information back to a controller for appropriate real-time action, in the latter. In this paper, embeddable fibers, embedding processes, and behavior of fiber-embedded metallic materials are reviewed. Particular emphasis has been given to embedding fiber Bragg grating (FBG) array sensors and piezo wires, because of their high potential to be used in nervous materials for structural health monitoring. Ultrasonic consolidation and laser-based layered manufacturing processes are discussed in detail because of their high potential to integrate fibers without disruption. In addition, current challenges associated with embedding fibers in metallic materials are highlighted and recommendations for future research work are set.

## 1. Introduction

Traditionally, metals and alloys are reinforced with fibers to produce metal matrix composites (MMCs) with increased strength and hardness that permitted their use in many industrial applications. In MMCs, the ductile matrix holds the reinforcement in position, and transfers the load to the strong reinforcement. On the other hand, the reinforcement imparts strength and stiffness to the matrix [1]. However, the growing demand for new generation of fiber-embedded materials, in the aerospace, military, and automotive industries, to enhance structural health monitoring has led to a shift from strengthening [1,2,3,4,5] to sensing [6,7,8,9,10,11,12] and nervous [13,14,15,16] applications. As a result, embedding fiber sensors in materials has attracted serious attention of researchers and become a new trend [17]. Such structures are usually termed “smart” [17] or “nervous” [16]. Smart materials are well established and characterized by smart behavior where the material senses some stimulus from its environment and reacts in a useful, reliable and reproducible manner [18,19]. On the other hand, “nervous materials” are an innovative class of materials characterized by feeling sense like humans and instantly reacting as a response to sensing. They are being developed where fiber sensors are volumetrically embedded and distributed emulating the human nervous system along with a network of actuators in key positions with respect to sensors. These materials are capable of feeling multiple structural and external stimuli e.g., stress, force, pressure, and temperature, while feeding information back to a controller for appropriate real-time action. The strain-stress state is developed in real-time with identified and characterized source of stimulus with optimized time response to retrieve initial specified conditions e.g., shape and strength. These materials will enhance personnel and public safety securing better reliability of products. Immediate applications of nervous materials are in aircrafts, cars, nuclear energy and robotics. Such materials will reduce maintenance costs, detecting initial failures and delaying them with self-healing [16].

The objective of this review is to provide a comprehensive description of embedding processes used to embed optical fiber sensors and/or actuators in metallic materials as a specific class of materials that has not been considered previously in the open literature. Composite materials were extensively reviewed [15,16]. This paper discusses the behavior of fiber-embedded metallic materials. Although, important embeddable fibers such as SiC and shape memory alloys (SMAs) are considered, to highlight their role in strengthening and sensing, respectively, particular emphasis has been given to embedding fiber Bragg grating (FBG) sensors in metals because of their high potential to be used for the development of nervous materials for structural health monitoring. Major embedding processes such as ultrasonic consolidation and laser-based layered manufacturing processes are discussed in detail because of their potential to integrate fibers without disruption. Published work on processing, structure, and performance of selected fiber embedded metallic systems is reviewed. Finally, current challenges associated with embedding fibers in metallic materials are highlighted and recommendations for future research work are set.

## 2. Embeddable Fibers

The selection of fibers to be embedded in metals and alloys is usually based on the expected added property and end application of the developed material. In this section, fibers are classified into three types, *i.e.*, conventional, smart, and optical; examples are given to highlight the role of the fiber in the developed material.

### 2.1. Conventional Fibers

**Figure 1:**
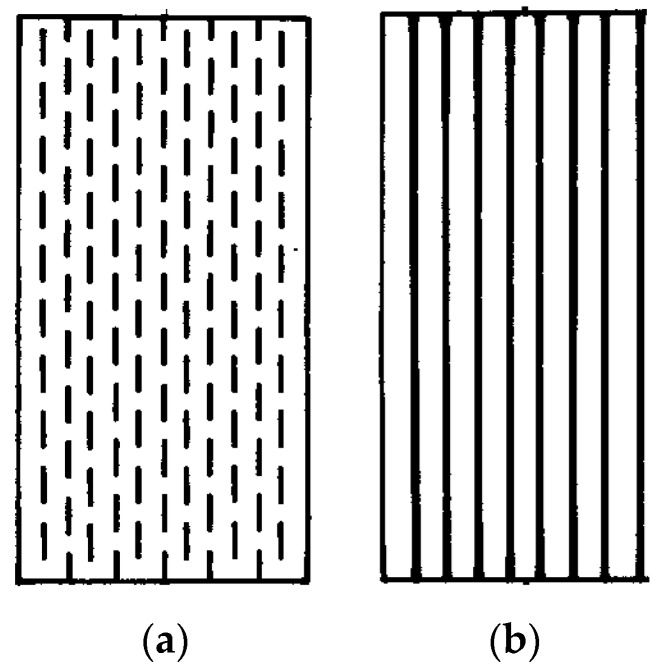
Composites reinforced with unidirectional (**a**) discontinuous and (**b**) continuous fibers.

Conventional discontinuous and continuous fibers, as shown in Figure 1, are used to develop metal matrix composites for structural applications. These fibers have high elastic modulus, high strength, and high stiffness. Amongst these fibers are silicon carbon fibers, which have excellent mechanical and physical properties, such as high strength, high modulus, high oxidation resistance and good compatibility with metals [20,21,22,23]. Their high strength and stiffness have made them a competitive material for unidirectional reinforcement of metal matrix composites for aerospace and automotive applications [24]. It was reported that reinforcing Ti-6Al-4V alloy with 35% by volume SCS-6 SiC fibers increased the stiffness in the longitudinal direction from 115 to 210 GPa [25]. In addition to strengthening the structure, they contribute to weight saving.

### 2.2. Smart Fibers

**Figure 2:**
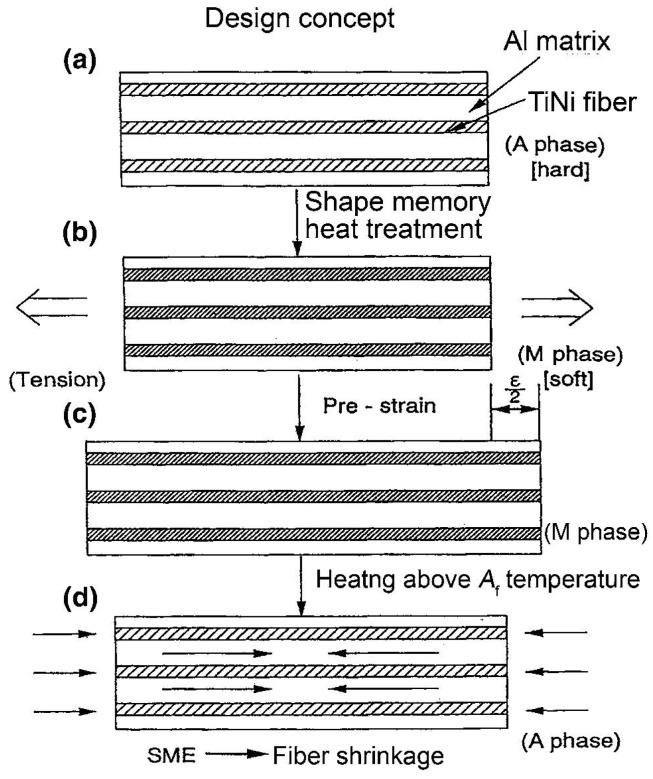
Design concept of shape memory alloy fiber-reinforced aluminum matrix Composite [28]. (**a**) As-fabricated composite; (**b**) Cooled composite; (**c**) Pulled composite; (**d**) Reheated composite.

Fibers made of shape memory alloys (SMAs) not only provide the structure with mechanical but also functional properties. SMAs are characterized with unique behavior such as shape memory effect (SME) and pseudo-elasticity (PE). SME refers to the material capability to recover apparently permanent deformation of up to 10% strain upon a modest increase in the material temperature. At high temperatures, SMAs behave pseudo-elasticity and may recover large deformations during mechanical loading-unloading patterns producing hysteresis [12,26,27]. SMA composites, fabricated by embedding SMA fibers into a host material, usually a composite, have shown potential applicability in a wide variety of smart systems and structures [12,27]. Design concepts of SMA fiber-reinforced aluminum matrix composite are presented in Figure 2 [28].

### 2.3. Optical Fibers

**Figure 3:**
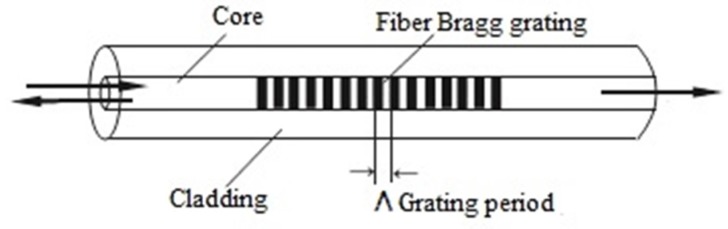
The structure of fiber Bragg gratings (FBGs).

An optical fiber is made of two layers *i.e.*, the core and the cladding, as shown in Figure 3. The light is guided through the fiber because of the slight refractive index difference between the two layers [29]. Sensors made of optical fibers [30] have excellent corrosion resistance and long lifetime. In addition, they are reliable, passive, and they do not require re-calibration overtime.

**Figure 4:**
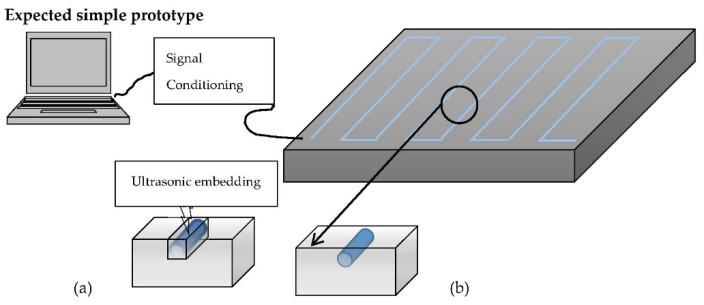
Design concept of aluminum embedded with FBG sensors: (**a**) glueing and (**b**) ultrasonic embedding.

In general, fiber optic sensors can be divided into three types: interferometric, distributed, and grating-based sensors. In interferometric based sensor, physical change in structure is reflected by the phase change of the two interfering light signals. Distributed fiber optic sensors can be divided into three categories: Optical Time-Domain Reflectometry (OTDR), Raman Optical Time-Domain Reflectometry (ROTDR) and Brillouin Optical Time-Domain Reflectometry (BOTDR) [16]. FBGs are one of the different optical fiber configurations [31]. They are usually photo-inscribed into a silica fiber for sensing purposes. FBGs are intrinsically sensitive to external stimulus such as temperature, pressure and strain; and yield a wavelength-encoded response, which can be recorded and processed [32]. Optical fibers have outstanding characteristics and properties such as lightweight, immunity to electromagnetic interference, stability, and little signal loss over very long distances. A summary of the mechanical and physical properties of different types of optical fibers that can be used as sensors and a list of measurands that can be monitored using optical fiber sensors were provided by Fernando [33]. Design concept of aluminum embedded with FBG sensors was already disclosed in [34]. Figure 4 shows an example of fibres embedding inside aluminum. It is worth noting that embedability in solid materials can be challenging, depending on the embedding depth and length needed for the fibers.

## 3. Embedding Processes

Embedding of fibers in a matrix mainly depends on the type of the fiber and matrix. Conventional discontinuous and continuous fibers are embedded in composites by casting, diffusion bonding, metal spray, or electrodeposition techniques. These methods have some limitations such as elevated processing temperatures, high cost of tooling, and limitations on geometrical complexity [2,3,4,5]. Smart fibers can be embedded in metals using techniques such as pressure casting, squeeze casting, pressure infiltration, hot pressing, spark plasma sintering, and ultrasonic consolidation [12]. As for optical fiber sensors although they were successfully embedded in polymer matrix composites [35,36,37,38,39], because of their flexibility, strength, and heat resistance; their use in metallic structures is very limited. This is because embedding fiber sensors in metals and alloys may lead to sensor degradation and sensitivity loss at high temperature [11]. Fortunately, embedding of fiber sensors in metallic materials is possible using ultrasonic consolidation (UC) [38] and laser based manufacturing processes (LLM) [10,39]. In the following section, UC and LLM processes are discussed in detail because of their high potential to integrate fibers without disruption. In addition, pre-embedding processes used to protect fiber sensors are highlighted.

### 3.1. Ultrasonic Consolidation

**Figure 5:**
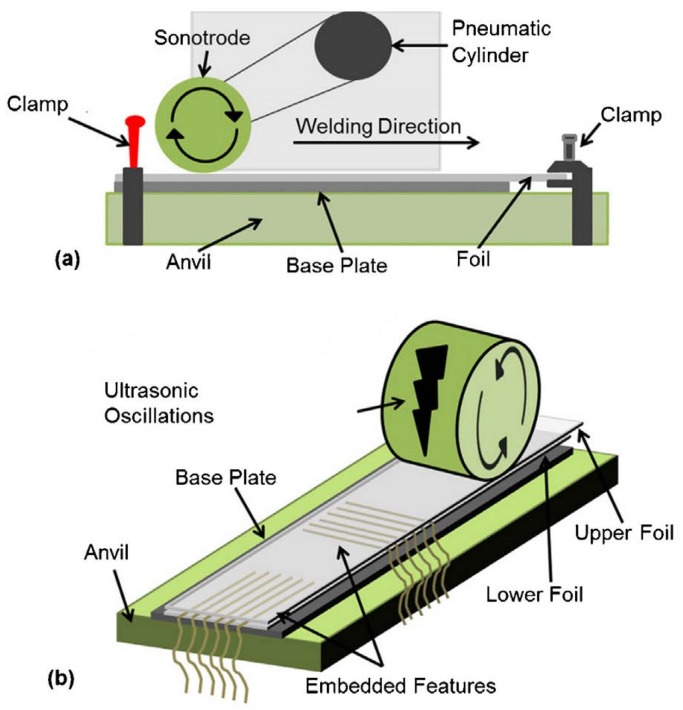
(**a**) Front view and (**b**) embedding features of ultrasonic consolidation process [40].

The ultrasonic consolidation (UC) process, Figure 5a, is a layer manufacturing process [40] that was invented by White [41]. It permits the deposition and bonding of metal foils layer by layer to create solid parts [40,42]. The process is characterized by two important features, the low temperature (≤50% of melting temperature) and the highly localized plastic flow during ultrasonic consolidation of the metal foils. These features allow certain components, prone to damage or sensitive to high temperatures to be embedded in a structure as shown in Figure 5b. The process was used to embed active [7,43], passive [44], and optical fibers [38] within aluminum matrices. In the UC process, the bond quality is controlled through the sonotrode clamping force, sonotrode oscillation amplitude at a given frequency, and sonotrode speed. In addition, the foil thickness, width and surface roughness influence the bond characteristics and quality [45]. The process permits extremely novel functionality to be achieved such as structures with embedded fiber sensors [46]. In the UC process high frequency (20–40 KHz) mechanical vibrations are used to bond (weld) metal foils and embed active/passive elements in the metal matrix.

**Figure 6:**
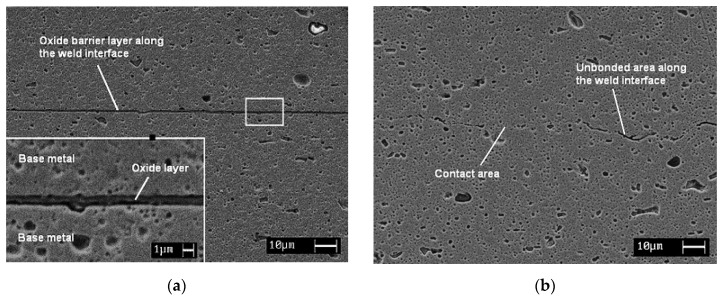
Micrographs of Al6061 foils (**a**) unprepared and (**b**) surface prepared welded using UC [47].

The UC process was successfully used by Kong and coworkers [47] to weld unprepared and surface prepared aluminum 6061 foils. The authors reported: (i) The existence of mainly Mg_2_O films, on the mating surfaces during the ultrasonic welding of Al6061 foils. These oxides may prevent the formation of metallic bonds; however, cleaning of foils prior to welding led to the formation of true metallic bonds. On the other hand, the oxides could be compacted together to form ceramic-based bonds. (ii) The possibility to have a high linear weld density and low peel load response. A linear increase in linear weld density was observed at high amplitude settings. However, the inverse was true for the peel test specimens produced at high amplitudes, where bonds could be weakened due to excessive strain-hardening and cyclic stressing of the contact points. (iii) The general process window for 100 μm thick aluminum 6061-T0 foils lies between 207 and 276 kPa contact pressure, 10.4–14.3 μm amplitude and 27.8–38.8 mm/s weld speed. Figure 6a shows micrographs of Al6061 unprepared specimen welded using UC. Approximately 500 nm thick oxide barrier layer along the weld interface was present. Micrographs of Al6061 prepared specimen welded using UC, Figure 6b, shows weld interface with contact points and oxides dispersed along the interface. The UC process was also used to embed different fibers in metallic structures as will be discussed below.

### 3.2. Laser Based Layered Manufacturing

**Figure 7:**
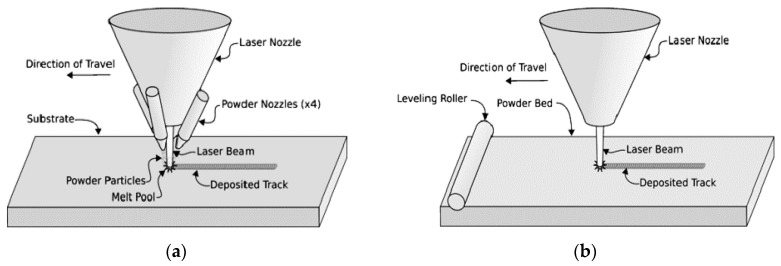
(**a**) Laser Engineered Net Shaping (LENS) and (**b**) Selective Laser Sintering (SLS) [49].

Physical parts are fabricated layer-by-layer using laser layered manufacturing (LLM) processes. The LLM process permits building parts that have traditionally been impossible to build because of their complex shapes or variety in materials. The process has several advantages including “building functional “smart” parts with sensors, integrated circuits, complete functional assemblies, and actuators placed within the structure and fully embedded. In particular, sensors embedded within the structural materials add intelligence to structures and enable real-time monitoring at some critical locations not accessible to ordinary sensors, which must be attached to the surface. Moreover, embedded sensors are also protected from damage caused by extraneous environmental effects. These sensors can be used to gain data for validating or improving designs during the prototype stage or to obtain information on the performance and structural integrity of functional components in service” [48]. Laser Engineered Net Shaping (LENS) and Selective Laser Sintering (SLS), as presented in Figure 7, are two types of LLM processes [49].

### 3.3. Pre-Embedding Processes

**Figure 8:**
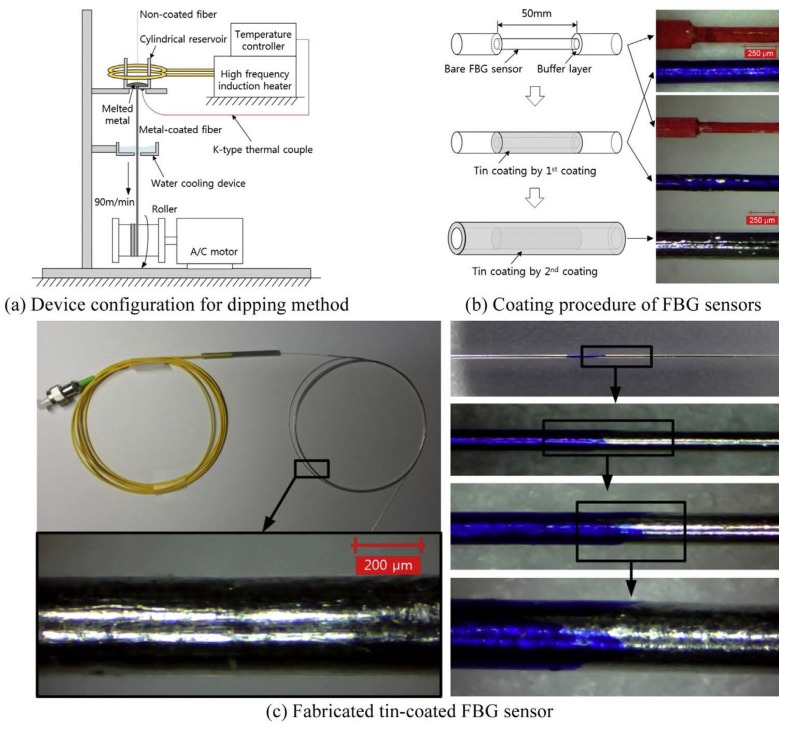
Coating procedures and fabricated sensors [50].

Conventional and smart fibers might be embedded in metallic materials without pre-preparation or special treatments. However, embedding optical fibers is challenging because the fibers need to be embedded in a metal structure that has a high melting temperature; and the thermal decay of UV-written gratings can start at a temperature of around 200 °C and accelerates at higher temperatures [10]. Therefore, optical fibers need to be protected during the embedding process to overcome the temperature and stress induced by the embedding process. In this case, the soft polymer coating on the optical fiber must be replaced by a protective metallic layer [50]. Figure 8 shows procedures to coat FBG sensors with tin by dipping method [50].

**Figure 9:**
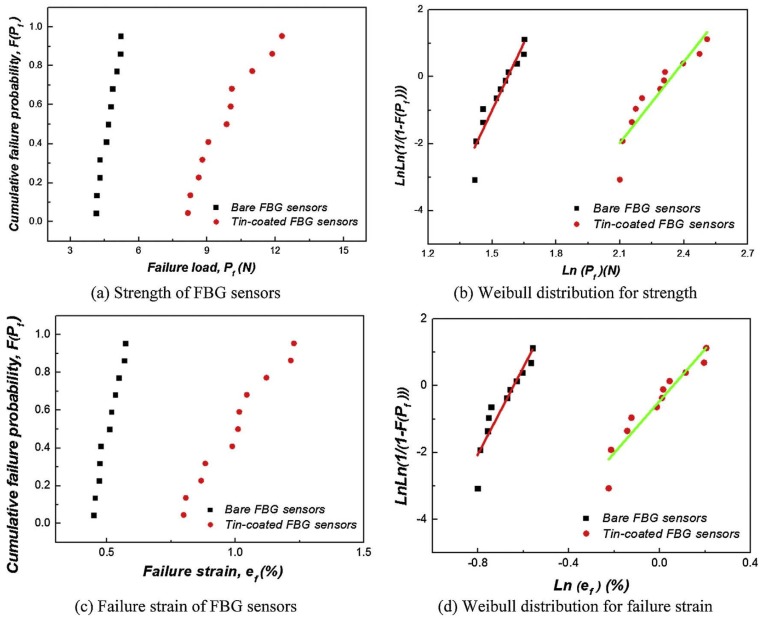
Comparison of failure parameters of bare FBG sensors and tin-coated FBG sensors [50].

Sang-Woo *et al.* [50] used cyclic loading tests to investigate the produced residual strain and the tensile failure strength of the tin-coated FBG sensors. They found significant amount of residual strain in tin-coated FBG sensors because of the elasto-plastic characteristics of the tin coating. However, the bare FBG sensors were almost strain free. Failure strength tests were performed and comparison of failure parameters of bare FBG sensors and tin-coated FBG sensors (Figure 9) were carried out. The authors found that tin coating was useful and contributed to protecting the sensors. The median failure strength of tin-coated FBG sensors was 111.8% greater than that of the bare FBG sensors. The Weibull modulus values were found to be 13.5 and 8.1 for the bare and tin-coated FBG sensors, respectively.

**Figure 10:**
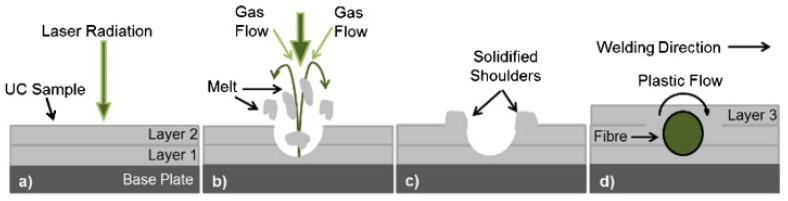
Channels created by manipulation of laser/material and assist gas interaction [40]. (**a**) Laser radiation; (**b**) Spatter/melt formation; (**c**) Channel creation; (**d**) Fiber embedding

Another method to protect fibers from the damage that may be caused by the increase in the UC sonotrode oscillation amplitude is to form a groove in the matrix material using a fiber laser, as shown in Figure 10, prior to fiber placement and subsequent UC. This permits the reduction of the required amplitude, and thus the necessary matrix plastic flow [40]. Channels were created in the samples using fiber laser irradiation (Figure 10a). By manipulating the resulting spatter/melt formation in combination with the assist gas the spatter/melt on either side of the channel boundaries could be distributed (Figure 10b,c), which would in turn reduce the required level of plastic flow during future fiber embedding (Figure 10d). The authors found that the most important parameters, for channel creation, were the laser power density and traverse speed. They concluded that laser is a promising tool for the creation of channels, which may allow embedding of high volume fractions of fibers in metals and alloys to produce metal matrix composites using the UC process [40]. It is worth mentioning here that embedding using UC method is only possible in foils to thin plates up to around 1 mm and embedding is at the surface. Some challenge arises to embed in depth and in larger plates compared to previous with UC method.

## 4. Materials Behavior

### 4.1. Strengthening Behavior

**Figure 11:**
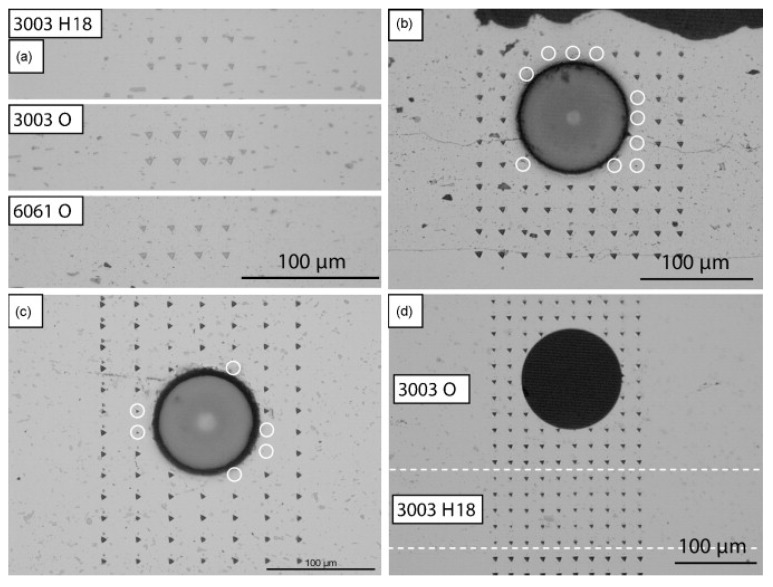
Microstructures of original foils and fiber embedded samples after nanoindentation: (**a**) original foils; (**b**) SiC fibers embedded in 6061 O; (**c**) SiC fibers embedded in 3003 O; and (**d**) a single mode optical fiber embedded in 3003 H18 and 3003 O (foil width direction: horizontal) [51].

The low strength and stiffness of metals has led to the development of fiber metal matrix composites where strong and rigid fibers such as SiC are embedded in ductile metal or alloy matrix such as aluminum. Analysis of the literature shows that SiC fibers were mainly embedded in aluminum alloys through ultrasonic consolidation to manufacture fiber-reinforced metal matrix composite (MMC) parts. Researchers had investigated the influence of process parameters on fiber embedment, characterized the interface between the fibers and matrix, and evaluated the mechanical properties of the developed materials. Li and Soar [51] successfully embedded SiC fibers in Al 3003 O and 6061 O alloys using UC process and investigated plastic flow of the alloys. They reported an increase in the hardness of the alloy, especially at regions close to the fibers. The authors found that work hardening followed the Hall-Petch relationship for both grains and sub-grains. The work hardening was attributed to the bulk plastic deformation during UC process with negligible effect from friction at foil/foil interface. The work hardening in the 3003 O matrix was found to be higher than that in the 6061 O matrix. Typical microstructures of original foils and fiber embedded samples after nanoindentation are shown in Figure 11 [51]. For the investigated alloys 3003 H18, 3003 O and 6061 O, the microstructure was composed of small particles dispersed in Al phases. The size of indentations in the original foils (Figure 11a) was in the following order: 3003 H18 < 6061 O < 3003 O. For the SiC fibers embedded samples (Figure 11b,c), some points in the matrices around the fibers (marked with circles) had much higher hardness than the Al alloys. However, for single mode fiber (SM) embedded samples, the indentations around the SM fiber had similar sizes and the indentations in 3003 H18 matrix were smaller than those in 3003 O matrix (Figure 11d).

In another investigation, the same authors [52] embedded continuous SiC fibers in an Al 6061 O alloy through ultrasonic consolidation at room temperature and determined the optimum embedding parameters through peel tests and metallographic analysis. In addition, they investigated the influence of the embedded fiber volume fraction and base metal thickness on the interface bond strength. The authors found that that friction at the consolidation interface is the main factor that influences interfacial bond strength. On the other hand, safe and full embedding of the fibers depended to large extent on the localized plastic flow around them. The optimum parameters for ultrasonic consolidation of SiC fibers in a 6061 O matrix were found to be: pressure from 135.1 to 176.3 MPa and amplitude from 6.8 to 12.3 μm for a traverse speed of 27.8 mm/s; and pressure from 135.1 to 176.3 MPa and amplitude from 8.4 to 14.3 μm for traverse speed from 34.5 to 43.5 mm/s. The consolidation strength in central areas of foil was higher than that at edges for monolithic samples and samples with one SiC fiber embedded due to a smaller load applied to the edges of the foil. However, for samples with more than five SiC fibers embedded, the consolidation strength in the central and edge areas became more even. The bond interface between SiC fibers and the 6061 O matrix was strong, and the failure of samples during fiber pullout tests was caused by the break of the SiC fiber itself. After embedding 0.8 vol % of SiC fibers in a 6061 O matrix, the peel strength of samples increased significantly up to certain volume fraction of embedded fibers, but there was a threshold for embedded fiber volume fraction at a specific parameter, over which weak bonding will form at foil/foil interfaces between fibers. The influence of base metal thickness on the peel strength was not significant, with an exception of samples with a base metal thickness of 500 μm.

**Figure 12:**
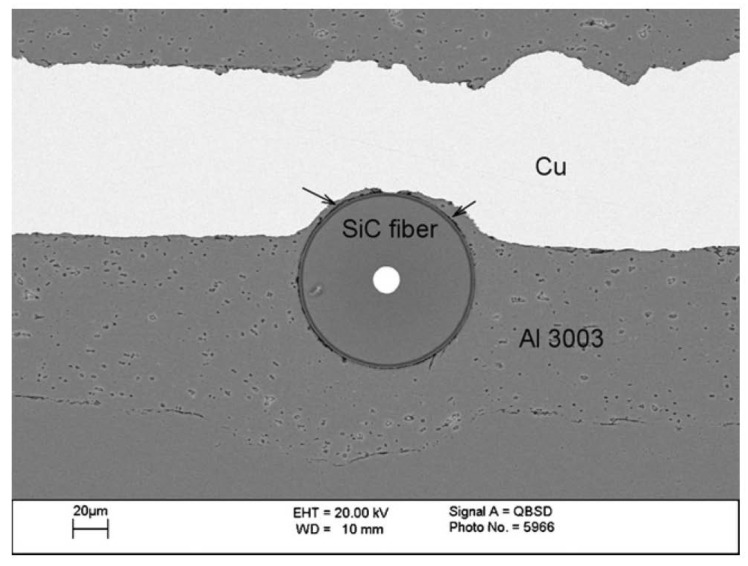
SiC fiber embedded between Al 3003/Cu foils. Note the aluminum flow around the fiber into the Cu side (shown by arrows) [53].

The microstructure of the interface of various ultrasonically consolidated similar (Al 3003/Al 3003 and Ni 201/Ni 201) and dissimilar (Al 3003/Ni 201, Al 3003/Cu) metallic materials was investigated by Yang and coworkers [53]. The authors found that none of the consolidated materials showed evidence of mechanical interlocking, localized metal melting, significant diffusion, or recrystallization at the weld interface. Bond formation between metal foils, at solid-state, was attributed to the atomic level forces across the nascent metal contact points. The authors concluded that the formation of bonds in ultrasonic consolidation mainly depends on the removal of surface oxide layers attained through frictional effects at the weld interface. On the other hand, interfacial plastic deformation facilitates intimate metal contact. The authors successfully embedded SiC fibers between Al 3003 and Al 3003, and Al 3003 and Cu matrices, as can be seen in Figure 12. The SiC fibers were mechanically entrapped in the matrix and diffusion or chemical reactions between fiber and matrix materials were not seen in the consolidated materials. The authors attributed the sound fiber embedment during UC to the plastic deformation of the matrix material.

The effectiveness of ultrasonically embedded SiC fibers in reinforcing Al 3003 matrix was investigated by Yang and coworkers [54]. The authors reported a significant improvement in peel strength and tensile strength of the consolidated parts. Samples reinforced with SiC fibers were found to have high peel and tensile strengths compared to unreinforced samples. However, the unreinforced samples showed better shear strength than those reinforced with SiC fibers. In another investigation [44], the UC process was used to embed SiC fibers in Al 3003 alloy [44]. The plastic deformation and flow of the matrix were found to cause sound fiber embedment. It was reported that the oscillation amplitude, welding speed, normal force, substrate temperature, and fiber orientation strongly affected the bond strength between the fibers and matrix. The authors concluded that “the following combination of parameters was found to produce the best fiber/matrix bond strength: oscillation amplitude of 20 μm, welding speed of 34 mm/s, normal force of 1700 N, substrate temperature of 149 °C, and fiber orientation of 45° [44]. Zhu *et al.* [55] investigated the phenomena occurring in the microstructure of the parts during UC process to obtain better understanding about how and why the process works. High-resolution electron backscatter diffraction was used to evaluate the influence of process parameters on the microstructure of AA6061 alloy and SiC reinforced AA6061 composite. The authors reported that ultrasonic vibration induced grain refinement, along the bond area, and affected the crystallographic orientation. Additional plastic flow occurred around the fiber led to the fiber embedding [55]. Sigma silicon carbide (SiC) fibers were also successfully embedded in alloys, without damage to the fibers, because of plastic flow of the matrix around the fibers [8].

### 4.2. Smart Behavior

**Figure 13:**
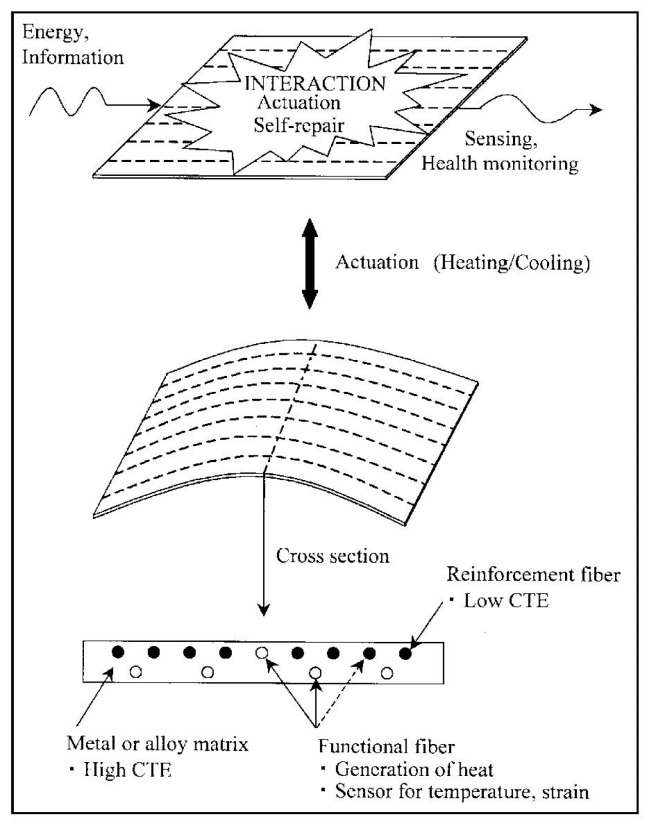
An active FRM embedded with functional fibers [57].

The use of sensors, actuators and micro-controllers with adaptive and/or intelligent functions is driven by the technology push towards “smart” systems [56]. These structures embedded with sensors and/or actuators are very attractive because of their potential use in damage detection, performance monitoring, noise reduction, vibration suppression, actuation, self-repair, and fabrication process monitoring [57]. Among materials used to build “smart” systems are fiber sensor embedded metals. Figure 13 “shows an active fiber-reinforced metal (FRM) with embedded functional fibers. The reinforcement fiber works as “bone” and the metal matrix works as “muscle.” Both are controlled by stimulation and energy transmitted through the functional fibers regarded as “nerve” and “blood vessel.” This materials system could have a variety of functions such as health monitoring and self-repair” [57].

In addition, fibers made of Shape Memory Alloys (SMAs) could be embedded in structures to provide not only mechanical but also functional properties. SMAs “are able to memorize or retain their previous form when subjected to certain stimulus such as thermomechanical or magnetic variations” [56]. They “can sense thermal or stress stimulus and exhibit actuation or some pre-determined response, enabling control of shape, strain, stiffness, natural frequency, damping and so on” [12].

Ni and Ma [12] reviewed the fabrication method, microstructure, interface reaction, modeling, and physical and mechanical properties of metal-matrix composites reinforced with SMA fibers as “intelligent materials”. They found that most of the published work was dedicated to the production of aluminum metal matrix composites reinforced with long nickel-titanium SMA fiber/wire using different fabrication methods as presented in Table 1 [12]. The authors concluded that the existence of the interfacial diffusion and/or reaction layer is a chemical result between the matrix and fiber and is very important information in the adhesion state of the interface. The fabrication method greatly influences the interface between the fiber and matrix. In addition, the authors pointed out to few issues such as phase stability, aging or degradation, and transformation hysteresis under particular constraints, which remain not well understood.

**Table 1:** Summary of the AMCs reinforced with long SMA fiber/wire and their fabrication methods [12]. AMCs: aluminum matrix composites. SMAs: shape memory alloys.

Matrix	Reinforcement (vol %)	Fabrication Method	Temperature/Pressure	Interface	Ref.
1100Al	NiTi_f_ (4–9)	Pressure casting	970 K/65 MPa	4 μm layer	[58,59]
1100Al	NiTi_f_ (3)	Hot pressing	843 K/200 MPa cool pressure	Unknown	[60]
AC4AAl	NiTi_w_ (2–4)	Squeeze casting	1,023 K/75 MPa	1.1 μm layer	[61]
1060Al	NiTi_f_ (20, 30)	Pressure infiltration process	973 K/30 MPa	Three layers Al_3_Ti, Al_3_Ni	[62]
6061Al	NiTi_f_ (19.5)	Vacuum hot pressing	813–823 K/2,000 kgf	Unknown	[63,64]
6082Al	NiTi_f_ (~20)	Hot pressing	806–833 K/25 MPa	Unknown	[65,66,67,68,69,70,71]
6061Al	NiTi_f_ (2.7–5.3)	Vacuum hot pressing	813–823 K/7–54 MPa	Al_3_Ti, Al_3_Ni	[72,73]
6061Al	NiTi_f_ (3.2–7.0)	Hot-press method	803–833 K/40–60 MPa	400 μm layer, Ti*_x_*Al*_y_*	[74,75,76]
2024Al					
Pure Al	NiTi_f_ (6, 20)	Vacuum hot pressed	873 K	Unknown	[77,78,79,80]
3003-H18	NiTi_f_ NiTi ribbon (5, 15, 20)	Ultrasonic consolidation	<573 K/<300 kPa	No	[7,8,81,82,83]
6061Al	NiTi (20)	Spark plasma sintering	633–873 K	Ni_3_Ti, Ti_2_Ni, Al_3_Ni	[84]

Hahnlen and Dapino [83] embedded NiTi fibers in Al 3003-H18 matrix using UC, investigated the strength of the fiber-matrix interface, and developed a constitutive composite model. They reported an average shear strength of 7.28 MPa for the interface. They found that the interfacial shear strength and the blocking force are not dependent upon length of the embedded NiTi elements, but the shear stress at the interface is inversely proportional to ribbon length. Examination of the NiTi-Al interface did not reveal metallurgical bonding or diffusion. The authors concluded that two additional avenues for strengthening the interface are possible: First, through promoting metallurgical bonding via oxide layer removal prior to embedding. Second, through the increase in a textured surface on the NiTi ribbon. This will not limit the composite failure to only failure of the interface.

Friel and Harris [85] successfully embedded NiTi SMA fibers into an Al 3003 (0) matrix using UC. They reported that the apparent bonding between fibers and the matrix was relatively weak and may be due to mechanical entrapment of the fibers within the matrix. In addition, the authors noticed lateral movement of the fibers, which may affect creating accurate, mechanically robust, fiber embedded metallic structures. The grains around the fibers were refined and work hardening was induced because of the embedded fibers. The authors concluded that this phenomenon “could be used to create MMCs that are very hard, compared to the raw processing foil, and/or the creation of functionally graded MMCs (*i.e.*, hard in desired areas). However, the UC process coupled with hard areas of sub-grain matrix can damage embedded SMA fibers through a combination of surface cracking and plastic flow”.

**Figure 14:**
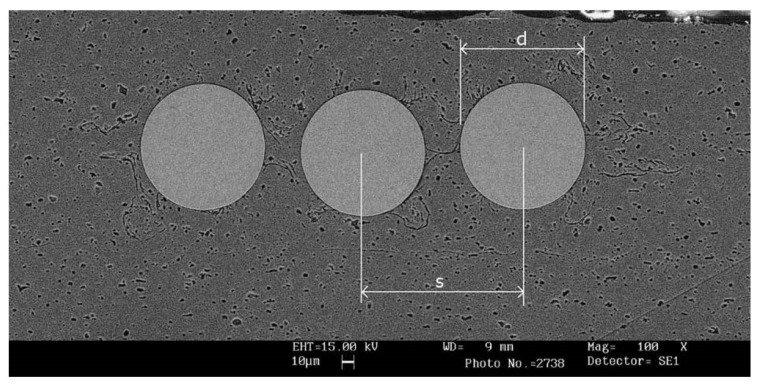
Scanning electron microscopy micrograph of SMA embedded specimen prepared at 27.8 mm/s weld speed, 10.4 μm amplitude and 276 kPa contact pressure (Keller's etched) [7].

Kong *et al.* [7] explored the possibility to use ultrasonic consolidation (SMA fibers embedded in aluminum alloy 3003 specimens) to fabricate adaptive composites as structural materials for advanced aerospace applications, which have the ability to measure and respond to external stimuli by adapting the structure accordingly, through embedded active or passive functional elements. They investigated plastic deformation of the matrix material around shape memory alloy (SMA) fibers, and bond quality, based on the microscopic observation and mechanical testing. The authors successfully produced laminate specimens, with full consolidation, within seconds, using low oscillation amplitude, low contact pressures (<300 kPa) and) and low temperatures (<25% of the fusion temperature). A typical SEM micrograph of SMA embedded specimen prepared at 27.8 mm/s weld speed, 10.4 μm amplitude and 276 kPa contact pressure is presented in Figure 14. The authors did not report evident chemical reactions at the fiber-matrix interface, and the fibers appeared to be physically and/or mechanically bonded to the matrix.

**Figure 15:**
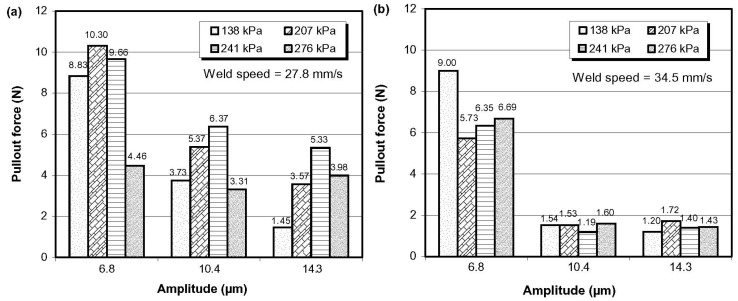
Fiber pullout test result of specimens consolidated at (**a**) 27.8 mm/s and (**b**) 34.5 mm/s, respectively [7].

The fiber resistance to pulling was function of the compressive and frictional forces applied to the fiber circumference. Unusual results were obtained from the pullout test, as shown in Figure 15. The authors attributed this behavior to the unique shape memory effect of SMA. At high amplitudes (10.4–14.3 μm), the SMA fiber transformed to its austenitic phase, as heating occurred, above the transformation temperature (70 °C), resulting in the fiber contracting in length and expanding in its circumference. This effectively reduced its resistance to pulling, during loading, as the SMA fiber returned to its martensitic phase, with a smaller fiber diameter, as compared to the cavity created in the deformed matrix. At a low amplitude (6.8 μm), no transformation took place resulting in a higher resistance to pulling. The authors concluded and showed that embedding SMA fibers in a material might change the thermal expansion rate if the system is heated above the fiber’s phase transformation temperature [7].

In another work, the UC process was used [8] to embed more ductile temperature sensitive and flexible SMA fibers without visible deformation or damage to the fibers similar to Figure 15. The authors concluded that metal-matrix and adaptive composites could be fabricated using UC process if fusion techniques become unavailable or a “cold” process is necessary for embedding.

### 4.3. Nervous Behavior

**Figure 16:**
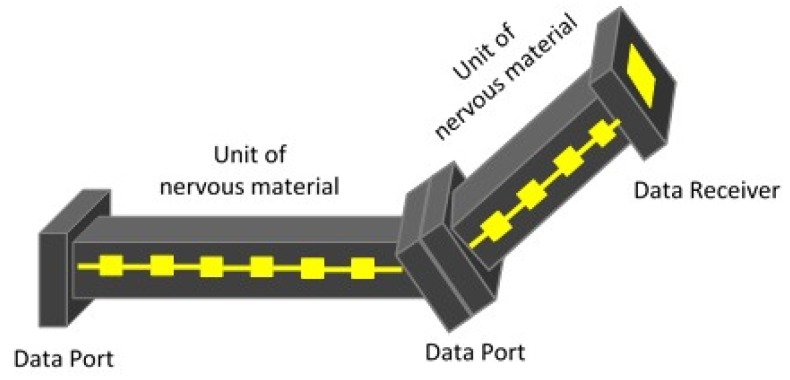
Sample of nervous material with embedded sensors and actuators with connectivity definition.

Modern structures using structural health monitoring require seamless distribution of sensors together with actuators both protected by the host material securing continuous measurement and correction. These materials constitute a new class of materials called nervous materials, which can behave and act as human skin and muscle combination coordinated with the sensory functions of the human nervous system to sense and react to external stimuli. In order to replicate such behavior in engineering structures, sensors need to be made part of the structure by concealing them within the material and hence protecting them from external harsh environment and also to develop a realistic picture of the material’s/structure’s internal response. Such a computed reaction is conveyed to the structure by embedded actuators over the volume of the part or distributed over just the necessary volume. Conceptually, the sensor and actuators are embedded and parts assembled with interfaces as shown in Figure 16.

**Figure 17:**
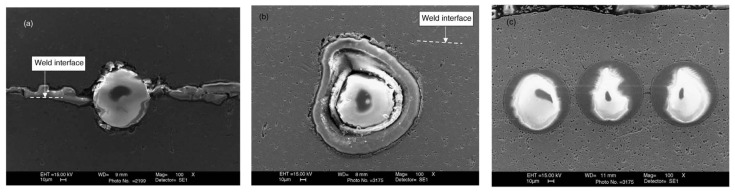
(**a**) Dispersal of polymer coating under full and partial load conditions; (**b**) coating remains but distorted in no load embedding and (**c**) full consolidation with polymer costing removed prior to consolidation [8].

**Figure 18:**
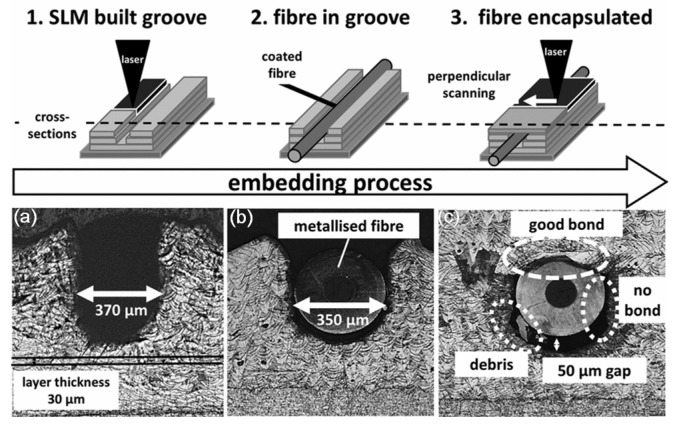
U-shaped groove built with SLM on SS 316 substrates (**a**), inserting Ni coated optical fiber inserted in the groove (**b**) and final encapsulation by continuing SLM process (**c**) [86].

Embedding of fiber optic sensors in metallic materials has been investigated by many researchers [8,86,87,88,89,90]. One of the objectives for this type of materials is to embed an FBG array optical fibers in multiple direction covering 3D space inside the material. Kong and Soar [8] investigated the embedability of optical fiber sensors made up of the fiber core (at 100 μm), glass cladding (at 140 μm) and an outer polymer coating (at 250 μm). The authors explored the possibility to embed the fibers with and without the polymer coating. They found that conducting experiments under “full” and “partial load” conditions, without removing the polymer coating, led to melting of the polymer coating, under ultrasonic excitation, as shown in Figure 17a. The authors noticed the deformation of the polymer coating under “no load” conditions (Figure 17b). The removal of the polymer coating led to full consolidation as can be seen in Figure 18c; and neither the glass cladding nor its core were cracked. However, chipping to the glass cladding was observed in some specimens prepared under extreme contact pressure of 345 kPa [8].

Havermann and coworkers [86] successfully embedded single mode optical fibers with thin nickel coatings (outer diameter ~350 μm) into stainless steel 316 components using bespoke laser based additive manufacturing technology as shown in Figure 18. The authors used Selective Laser Melting to incorporate U-shaped grooves in SS 316 components with dimensions suitable to hold nickel coated optical fibers. Coated optical fibers containing fiber Bragg gratings for strain monitoring and temperature sensing were placed in the groove. The embedding was completed by melting subsequent powder layers on top of the fibers. The authors reported a strong substance-to-substance bond between coated fiber and added SS 316 material. Temperature and strain cycling of the embedded sensors demonstrated the ability of gratings to survive the embedding process, and act as sensing elements in harsh environments. *In-situ* strain and temperature measurements from within the component were demonstrated for high dynamic stress levels and elevated temperatures (<400 °C). The gratings optical properties were maintained during the embedding process, enabling *in-situ* measurements of strain and temperature changes. Repeatable strain measurements with high dynamic stress levels have been demonstrated [86].

Maier *et al.* [87] used additive manufacturing to embed sensors in metallic structures. These embedded sensors would be capable of operating at extremely high temperatures by utilizing regenerated fiber Bragg gratings and in-fiber Fabry-Perot cavities [87]. Taylor *et al.* [88] and Lee *et al.* [89] used casting to embed a fiber Fabry-Perot Interferometer into aluminum. Baldini *et al.* [90] incorporated gold-coated optical fibers into titanium matrix composites produced by arc spraying. Other researchers [6,91] cleaned the optical fibers and substrates, and then sputtered a conducting thin metallic film on the fibers. This was followed by electroplating the fibers with a protective layer of Ni layer, and finally laser cladding the treated fiber with a stainless steel layer, which permitted sound embedding.

Li and coworkers [92] used laser assisted shape deposition manufacturing (LASDM) methodology to embed FBGs in stainless steel structures. They followed the same steps as reported in [6,91]. The authors found that cleaning the substrate prior to nickel deposition leads to embedding with full contact between the silica fibers and the Ni matrix. The electrolytically deposited nickel had a microstructure with a grain size of 150 nm before laser cladding. The deposition of stainless steel using LASDM led to grain growth and the grain size reached large values at 1.5 mm from the stainless steel/Ni interface. Ni grains remained very fine with grain size within the range 1 to 10 μm in the immediate layer surrounding the fibers. The embedded fibers significantly affected the transmitted light when the thickness of the nickel layer was less than 1.5 mm [92].

**Figure 19:**
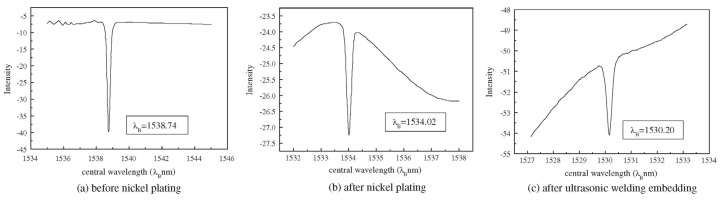
The Bragg peak of a FBG before Ni coating, after Ni plating, and after embedding [93].

Li and coworkers [93] explored the possibility to embed metal-coated FBG in similar and dissimilar metals, *i.e.*, copper and aluminum. In addition, they investigated the influence of protection methods on the embedability of the fibers. They used bare fibers as well as fibers coated using chemical plating chemical-electroplating fibers. Since coating may affect the performance of the FBGs, the authors examined the thermal and strain sensing characteristics of the embedded FBGs. Typical Bragg peaks of a FBG before nickel coating, after nickel plating, and after ultrasonic welding embedding are shown in Figure 19 [93]. The authors noticed the preservation of the Bragg peaks form after the coating and consolidation. However, they reported a shift down of the Bragg peak wavelength by about 4.72 nm because of nickel chemical-electro plating. The peak was further shifted down by 3.82 nm because of ultrasonic consolidation. These shifts were attributed to the fact that the fiber is subjected to axial compression and thermal contraction of aluminum because of Ni deposition during plating and thermal contraction of aluminum substrate during cooling, respectively [93].

**Figure 20:**
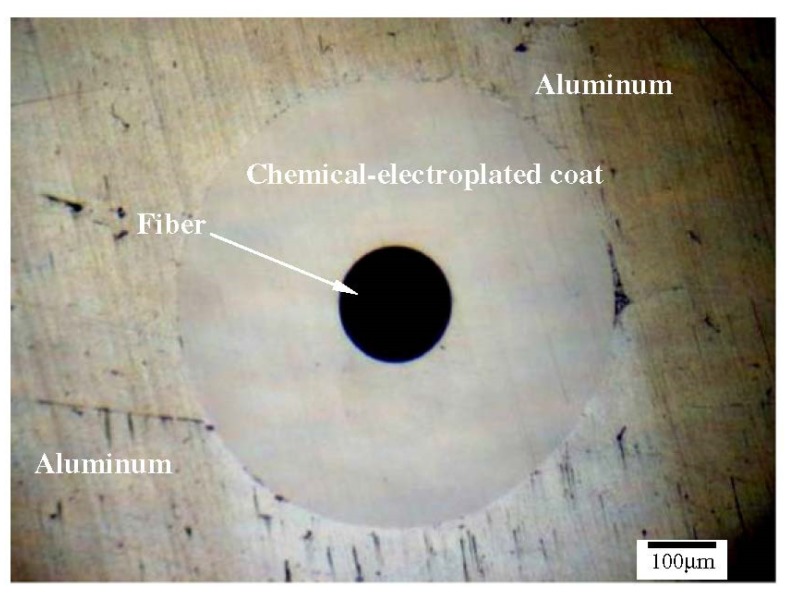
The aluminum/chemical-electro plated fiber/aluminum [93].

The authors reported that it was not possible to embed the bare fibers in Cu because of its high hardness. Similarly, embedding of the chemical plated fiber failed because of the welding pressure, vibration and friction. However, the chemical-electroplating method yielded a well-protected FBG. The fiber was successfully embedded in aluminum as shown in Figure 20. Protection of the fiber enhanced its temperature sensitivity to about twice of the unprotected fiber. The difference in temperature sensitivity recorded before and after embedding in Al was attributed to the different thermal expansion coefficients of the aluminum (13 × 10^−6^/K and 23 × 10^−6^/K) and the electroplated nickel layer (23 × 10^−6^/K). The embedded FBGs in the welded structure were not destroyed when tensile load ranging from 0 to 40 N was applied. The wavelength and the load showed a linear trend [93]. The authors evaluated the response of the FBGs to the applied tensile load. The wavelength shift for both FBGs followed a linear trend. This was attributed to the linear characteristic of the FBG and linear structural properties of the metal coating. The authors experimentally confirmed that when the tensile load (0–40 N) is applied on the embedded FBGs, FBGs remained in good condition because any debonding may cause deviation from linear trend of the wavelength shift [93].

Masurtschak and coworkers [40] explored the possibility to create microchannels in Al 3003-H18 samples using fiber laser in order to make fiber layout patterns, which may reduce plastic flow and ease fiber embedding in UC process. The effect of laser power, traverse speed and assist gas pressure on channel formation was investigated. The authors found that accurate melt distribution and channel geometry in the micrometer range could be easily obtained using multiple laser passes. The most influential parameters for channel creation were found to be the laser power density and traverse speed. The Gaussian profile of the produced channels was suitable for secure positioning and embedding of circular profile fibers. However, their width was difficult to control, which may lead to the movement of fibers within the channels during ultrasonic consolidation [40].

Cutting tools with embedded FBG sensors were successfully developed using laser-based layered manufacturing processes [10]. The embedding process consisted of low temperature laser microdeposition of on-fiber silver thin films followed by nickel electroplating in a steel part. A microscale laser-based direct write (DW) method, called laser-assisted maskless microdeposition (LAMM), was employed to deposit silver thin films on optical fibers. To attain thin films with optimum quality, a characterization scheme was designed to study the geometrical, mechanical, and microstructural properties of the thin films in terms of the LAMM process parameters. To realize the application of embedded FBG sensors in machining tools, the electroplating process was followed by the deposition of a layer of tungsten carbide-cobalt (WC-Co) by using laser solid freeform fabrication (LSFF). An optomechanical model was also developed to predict the optical response of the embedded FBGs. The linear response of the embedded sensor showed the integrity of the layers and the absence of cracks, porosity, and delamination, which was also confirmed by microscopic imaging [10].

Li and coworkers [94] proposed a new technique for embedding FBG sensors into metals such as Ni. The technique involves low temperature processes, magnetron sputtering and electroplating. The optical fiber can be continuously electroplated into a thicker metallic layer or embedded into other metallic structures by high-temperature processing. The embedded FBG sensor had an accuracy of about 2 °C [94].

Havermann and coworkers [95] used bespoke laser based additive manufacturing technology to embed single mode optical fibers containing high reflectivity Bragg gratings into stainless steel components. The gratings survived the embedding process and acted as temperature or strain sensors. At higher temperatures up to 500 °C, discontinuities in the gratings response were observed [95].

Li and coworkers [96] solved the problem of fiber Bragg grating (FBG) sensor protection and embedding in metal through nickel coating the FBG using chemical and electric plating method. The metallized FBG was then embedded into 42CrMo steel successfully and a smart metal part was acquired. Experimental results showed the FBG was protected successfully with the nickel layer, and there were no defects at the FBG/nickel layer interface. The heat did not wreak the FBG during soldering process. Temperature monitoring results showed the temperature sensitivity of FBG was increased twofold after metallization and soldering, and the temperature change was linear with reflection wavelength [96].

Li and coworkers [97] embedded fiber sensor in copper and aluminum foil using ultrasonic welding of T2 copper and 1100(L4) aluminum. Bare fiber, fiber with chemical plating coat and fiber with chemical-electro plating coat were used. They authors concluded that aluminum foil could be used for embedding the fiber sensor. Chemical-electro plating method could be used as the protection method for the fiber sensor. The average embedding strength was 45 N and the light intensity loss was 0.22 dB [97].

**Figure 21:**
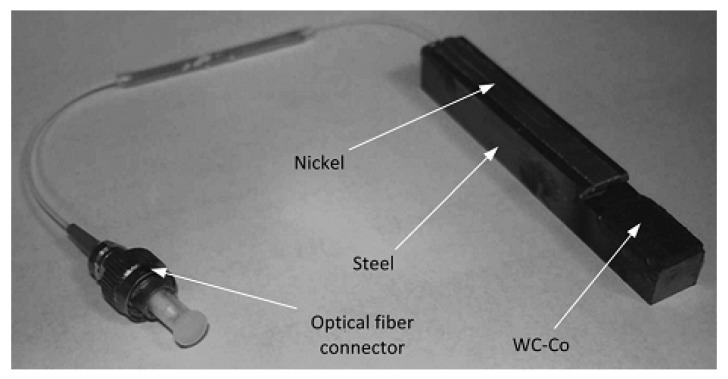
Prototyped sample with metal-embedded FBG using laser-assisted maskless microdeposition (LAMM) and electroplating [10].

Alemohammad and Toyserkani [10] developed a new technology for embedding FBGs in metal cutting tools where a hard machining surface, made of tungsten carbide-cobalt (WC-Co), was deposited on the steel part. Figure 21 [10] shows prototyped sample of cutting tools with embedded FBG sensors manufactured using laser-based layered manufacturing processes and electroplating. The authors claimed that the embedded FBG could act as a load and temperature monitoring device when the machining tool is in service.

**Figure 22:**
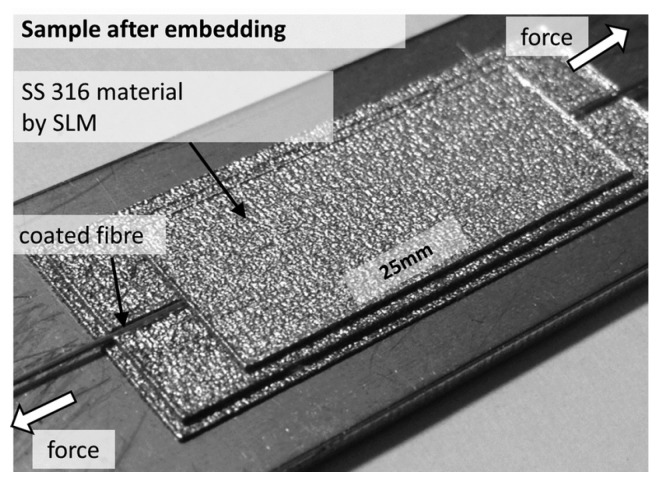
Image of steel sample with embedded FBG for strain testing [86].

Havermann and co-authors [86] demonstrated the feasibility of embedding FBG into SS 316 components by using powder bed based selective laser melting (SLM) for strain testing as shown in Figure 22. The authors reported repeatable strain measurements with high dynamic stress levels.

## 5. Current Challenges

Fiber sensors and/or actuators were successfully embedded in thin plate metals at the surface layer, to create “smart” structures; however, the embedding process of optical fibers and piezo wires in metals to create “nervous” materials is in its infancy since this aspect needs an in-depth embedding of FBG array fiber optic sensors in multiple directions over the volume of the parts. The sensor placement in multiple directions needs more investigation to avoid neutral areas loose interface with the host material and hence be able to report useful information. The other challenge resides in the fact that the optical fiber sensor is highly delicate and brittle, and would be damaged during lengthy embedding processes to cover dimensions of the part. Hence, embedding FBG sensors in metallic structures remains a challenging task. Furthermore, coating of FBG sensors with metallic films proved to be useful to protect them from damage when measuring high temperature and improve their performance in harsh environments, while it may alters the sensor sensitivity. Actuating bulky structure with embedded actuators is challenging at the moment depending on the actuation capacity, volumetric coverage of the embedded actuators and stiffness of the material.

## 6. Future Directions and Applications

Sensors, actuators and micro-controllers with adaptive and/or intelligent functions have become fundamental components of new intelligent, nervous materials that can also adapt to their surrounding changing environment. Hence, structural shapes become sensitive, controllable and active reacting to external effects. It is hence required to develop miniaturized sensors not only able to be embedded in the structure without any loss of structural or functional performance *i.e.*, extra weight, strength degradation, but also to keep their integrity while embedded. The existing structures are metallic in nature but needing to be smart enough to inform about any constraint and possible unexpected failures. Special embedding techniques for two and three dimensions and associated problems need to be addressed in small and large part volume. The future generation of materials is envisaged to behave more on biological analogy to human nervous system with distributed embedded sensor array and actuators architecture developing a biologically inspired nervous system. Embedding of optical fibers and piezo fibers in metals within functional geometries to create “nervous” materials is believed to be a cutting-edge research topic. With successful development of these materials, it will be potentially possible: (1) to enhance personnel and public safety, and secure greater reliability of products; (2) to reduce maintenance cost; (3) to detect an early failure mode in real time; (4) to monitor a product that has a large and/or sensitive structure e.g., aircraft or nuclear power plant; and (5) to compensate for any deformation and prevent failure (self-healing).

The new generation of materials that feels and reacts by reshaping with high performance to external effects and be able to improve functional characteristics e.g., strength, aerodynamics, delaying failures are very desirable. Future materials may inform about their weakness and advanced wear level. Light sent through fiber optics is able to generate acoustic vibrations. The corresponding pitch can change with temperature and hence temperature mapping can be built around the fiber optics network. Several measurands can be monitored through fiber optics. With this feedback and required self-reaction, these materials will couple sensing, actuation, computation, and communication.

Materials with embedded FGBs or equivalent sensors will be extensively used in structural measurements, failure prognosis, thermal measurements, and pressure monitoring to cite a few. In particular applications, selected metal parts within structures embedded with sensors and/or actuators can be used as key element, e.g., machining tools, and aerospace and automotive industries.

For difficult to reach or independent parts, powering the sensors and actuators can be challenging. Hence, power harvesting from within the part can constitute a fundamental solution if the level of produced power through vibration, ambient radio frequency (RF), *etc.* is sufficient.

**Figure 23:**
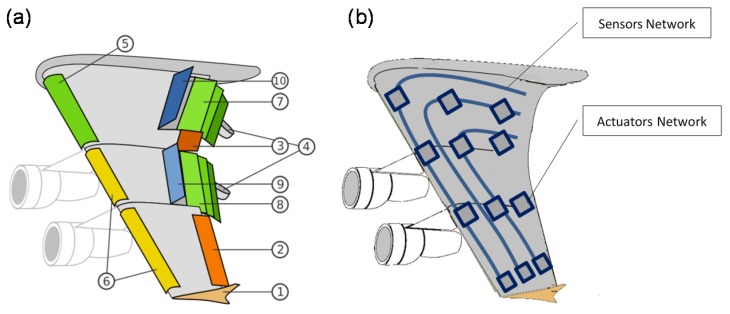
(**a**) Current airplanes wing with flaps and brakes and (**b**) expected nervous seamless wing.

For example, the current airplane wings will have all the dozen of actuators, air brakes and flaps removed and replaced only by a wing with seamless embedded sensors and actuators to actuate flexibly the wing as required by the continuous measurements taken and analyzed by the on-board controller as shown in Figure 23.

## 7. Conclusions

A comprehensive review of embeddable fibers, embedding processes, and the behavior of fiber-embedded metallic materials was presented. The following conclusions can be drawn from the present review:

1. Smart fibers were successfully embedded in metals, using a wide range of techniques, to create smart materials but at subsurface level only.
2. Ultrasonic consolidation and laser-based layered manufacturing processes remain the most suitable techniques to embed optical fibers in metals. Some laboratory tested examples proved to be successful.
3. Pre-embedding treatments might contribute to the protection and facilitate the embedding of optical fibers without much damage of the fibers.
4. The embedding process of optical fibers in metals is in its infancy; and it is believed to be a cutting-edge research topic when considering most aspects securing in-depth and volumetric embedding of lengthy fiber optics.
5. Successful embedding of optical fibers and actuators array in metals without disruption would pave the way for further development of “nervous” materials.
6. There is a potential to modify and upgrade embedding techniques, used for embedding smart fibers, and to extend their use for the development of nervous materials if the discussed challenges are addressed.
